# New insight into the swimming kinematics of wild Green sea turtles (*Chelonia mydas*)

**DOI:** 10.1038/s41598-022-21459-y

**Published:** 2022-10-31

**Authors:** Nick van der Geest, Lorenzo Garcia, Roy Nates, Daniel A. Godoy

**Affiliations:** 1 BioDesign Lab, Auckland University of Technology, Auckland, 1010 New Zealand; 2 Blue Planet Marine, Canberra, Australia

**Keywords:** Marine biology, Mechanical engineering

## Abstract

Biomechanically, sea turtles could be perceived as birds of the ocean as they glide and flap their forelimbs to produce the necessary forces required for locomotion, making sea turtles an interesting animal to study. However, being an endangered species makes studying the sea turtle's biomechanics a complex problem to solve, both technically and ethically, without causing disturbance. This work develops a novel, non-invasive procedure to develop full three-dimensional kinematics for wild sea turtles by filming the animals in Australia's Great Barrier Reef using underwater drones without disturbing them. We found that the wild animals had very different swimming patterns than previous studies on juveniles in captivity. Our findings show that the flipper goes through a closed-loop trajectory with extended sweeping of the flipper tip towards the centre of the carapace to create a clapping motion. We have named this the “sweep stroke” and in contrast to previously described four-stage models, it creates a five-stage cycle swimming locomotion model. The model presented here could lead to a better comprehension of the sea turtle propulsion methods and their fluid–structure interaction.

## Introduction

The locomotory biomechanics of sea turtles share similarities with birds, given that they glide and flap their forelimbs to produce the necessary forces required for locomotion. In fact, in recent studies, phylogenomic analyses has shown turtles as the sister group of birds^1^. However, unlike birds who require lift generation to remain in the air, sea turtles can adjust their buoyancy to become almost entirely neutrally buoyant^2^. Thus, lift generation is not wholly required as their buoyant force may be sufficient. The forward thrust generated by turtles to swim has been shown to almost entirely occur during the downstroke, using their cambered aerofoil-shaped flippers^3,4^. The downstroke has been previously described as twice as fast as the upstroke, typically producing a figure-of-eight pattern^3–6^. Prior research has typically illustrated that sea turtles apply an angle of attack (AOA) of their pectoral flippers^3,4^ so the animal can generate the necessary hydrodynamic loads for swimming. However, this is not entirely accurate as the flipper generates a variable AOA across their wingspan via twisting of the forelimb.

Sea turtles are migratory species with end-to-end journeys of thousands of kilometres^7–9^. Having a mathematical representation of their swimming locomotion would greatly help answer whether or not the sea turtle's swimming style is a factor in their migratory success. Previous studies have applied simplified two-dimensional models of turtle inspired swimming styles^10–12^. However, hydrodynamic features that influence propulsion are highly dependent on the kinematics^13–15^, which suggests that while the research done to date provides a rudimentary approach to help describe the propulsion mechanism, it does not contain all the related factors. Various studies have illustrated the natural sea turtle locomotion^3,4,16–20^. However, these studies often collected data on young juveniles held in captivity without describing the patterns in a mathematical form. Therefore, the natural swimming description of wild sea turtles is still unknown, along with understanding if it differs from a juvenile in captivity.

Developing a mathematical model to describe the turtle's natural flipper patterns is tremendously complex for several reasons. Among the difficulties, the most complex issue comes from producing a data collection from within their natural habitat to ensure their surroundings do not enforce an abnormal swimming pattern. The current state of the art techniques, such as X-ray Reconstruction of Moving Morphology (XROMM)^21,22^, requires a unique set up that would be impractical to impossible to set up in a marine environment. Not only are techniques like this impractical, but receiving animal ethics approval to put an endangered species into a lab to study its biomechanics creates further hurdles.

This paper describes a non-invasive process to film sea turtle swimming patterns and forelimb twist through a video camera attached to a remotely operated underwater vehicle (ROV). The films formed the basis for generating 3D mathematical biomechanical pattern reconstruction. Filming sea turtles with an ROV has been done before^23,24^. However, to the best of our knowledge, this work is the first report of collecting and analysing turtle flipper kinematics using an underwater ROV. This paper also includes developing and applying an original data processing method to uncover the full 3D kinematics of the turtle flipper movement.

## Methods

### Data collection

#### Filming procedure and locations

**Figure 1:**
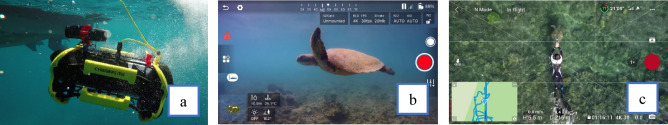
(**a**) ROV with external camera launched from the rigid inflatable vessel. (**b**), ROV user interface showing sea turtle applying general swimming routine, (**c**) Sea turtle swim speeds from GoPro GPS, with snorkelling assistance from the top. The photograph was taken at Fitzroy Island using the DJI fly IOS application with a DJI mini 2. https://www.dji.com/nz/downloads/djiapp/dji-fly.

To collect detailed data on the kinematics of wild turtles, it was decided to film them in their natural environment. Filming occurred over a three-week program at Australia's Great Barrier Reef, with the approach slightly altering depending on location. All permits and animal ethics approvals were obtained from the Australian Government Marine Park Authority (reference number G21/45637.1), with all methods performed in accordance with guidelines and protocols approved. Only green sea turtles (*Chelonia mydas*) were observed during this time. The first half of the data collection was accomplished at Fitzroy Island, Cairns, Australia, using an ROV shown in Fig. 1a deployed from the shoreline over shallow fringing reef. On average, four dives were made per day, each lasting approximately 30 min, recording the entire dive duration. The camera used was programmed to save the footage at 2 GB segments to minimise the chances of a total data loss. The ROV, on average, would travel 25–75 m from the beach until turtles were observed.

A second location was established at Heron Island, Queensland, Australia, and the filming procedure was assisted with a rigid inflatable vessel to launch the ROV seen in Fig. 1a. Data collection follows the same routine described for the first location. However, the vessel was the launch platform enabling a higher degree of habitat and depth options to ensure a full suite of natural swimming behaviours were captured.

At all times, during the approach to a turtle, a distance was kept of approximately 5 m to ensure the animal was undisturbed and continued with its usual swimming pattern. In general, the turtles showed no interest in the ROV and would continue the locomotion as usual. On rare occasions, we found that some turtles even showed interest in the ROV and approached it for closer inspection out of curiosity. The ROV was set in hover mode to allow the animal to interact when this occurred. Therefore, the recording was only made if the turtle was observed swimming in its general swimming pattern. This pattern was identified with the turtle producing complete oscillations of its pectoral fins with its hind flippers tucked in and pointing back to lower its pressure drag, captured in Fig. 1b. Filming was done from the side, front and rear viewpoints with the ROV following the turtle, matching its swim speed to keep the turtle centred on the camera lens. This process proved to work well for filming from the sides; however, the turtles would often adjust their swim patterns when attempting to film from the front or rear. Turtle swim speeds were estimated using a surface snorkeler holding a camera with GPS by following the turtles from the surface, as illustrated in Fig. 1c. Swimming speeds of approximately 0.2 to 1 m/s were observed and correlate well with previous research values^25–29^.

#### Equipment specification and settings

The ROV used (Chasing M2) was equipped with a 200 m long tether connected to a handheld transmitter. The camera on the ROV was never used to film and only used for feedback to the operator. The system has a maximum dive depth of 100 m, however, it was only used to 2–30 m. The system is equipped with 8 EDF thrusters to allow 6 DOF allowing the ROV to manage ocean currents with speeds up to 1.5 m/s. A 3D printed adaptor was manufactured to enable an extra camera to be mounted to the ROV chassis. The camera (Paralenz, Vaquita) was set up to film at full HD 1080P, 240 frames per second, allowing smooth slow-motion footage. The footage was stored to a 128 GB SanDisk extreme pro micro SD card. The camera was selected as the best option available because its slim rugged aluminium body did not require a waterproof case. Additionally, it is equipped with a distortion-free lens and higher frame rate compatibility compared to other cameras available at the time.

### Data processing

#### Data preparation

**Figure 2:**
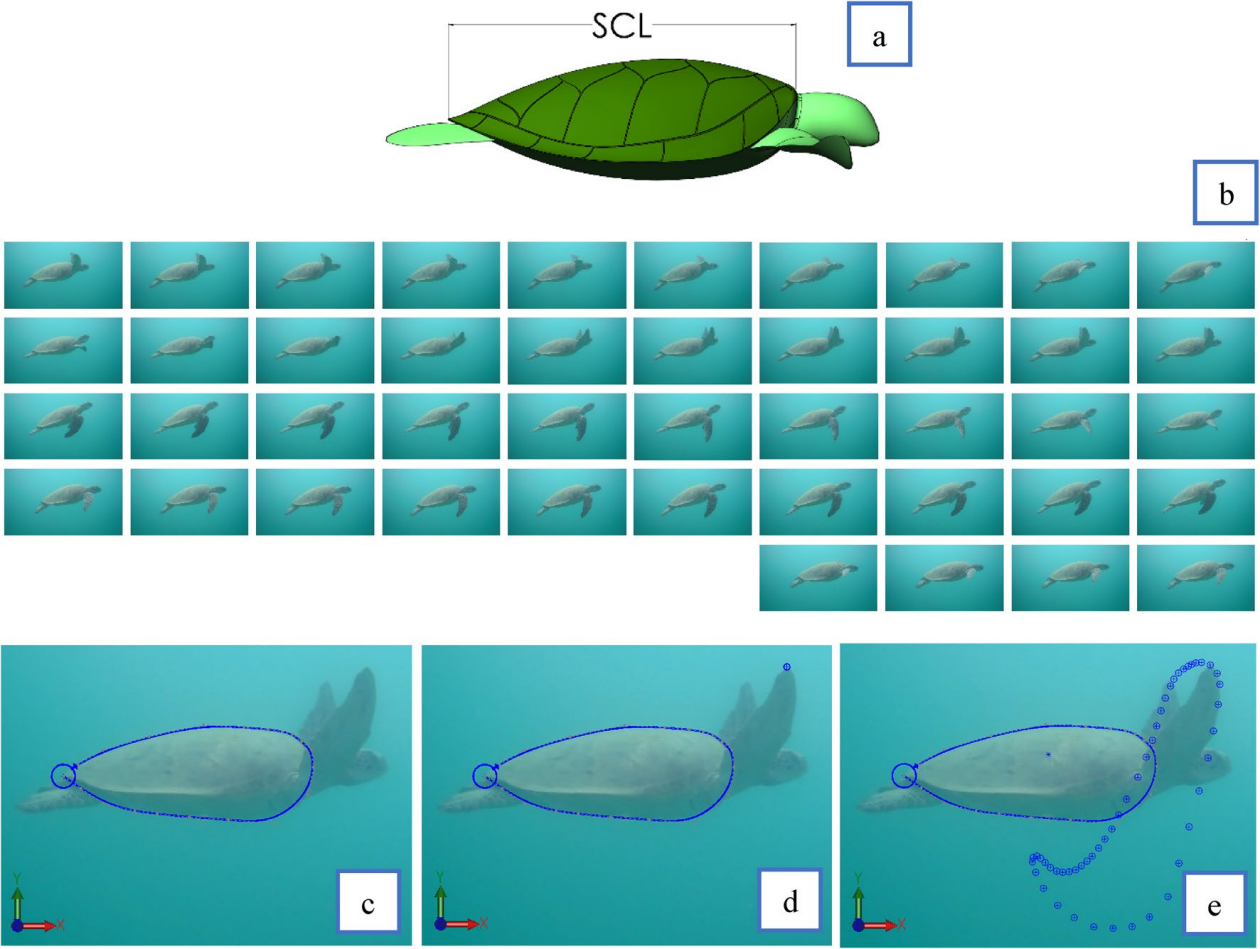
Data preparation illustration. (**a**) Sea turtle straight carapace length measurement (**b**), photos of turtle at 0.1-s intervals; (**c**), 2D spline around turtle carapace to serve as reference geometry; (**d**), Datapoint placed at flipper tip; (**e**), Assembly of data points with reference to carapace and time.

The challenge in this work was to extract the absolute motion of the flipper with respect to the straight carapace length (SCL). The SCL illustrated in Fig. 2a was used as the reference frame of motion due to it being a rigid body and thus not changing in size. Side view footage was prioritised due to its more accessible collection, and additionally, this orientation allowed for a clear sight of the turtle's flipper tip for all values of time. The film footage was edited in software (VideoPad Professional, NCH Software). This process involved snipping and exporting the content into individual images that matched the film frame rates. Thus allowing detailed photos of the flipper position with respect to time. Using a CAD system (Solidworks 2019 SP5), the photos were imported using the "sketch picture" feature and sorted in the design space at 0.1 s time intervals shown in Fig. 2b. A single image was selected for creating a 2D spline around the turtle carapace. This 2D spline was then placed over each image to ensure each image was not distorted and suitable for data collection seen in Fig. 2c. This process also gave a reference coordinate system to ensure the data could be accurately knitted together. After confirmation that each frame was appropriate, a data point could be placed on the turtle's flipper tip for every image as per Fig. 2d. An assembly could then be made off each flipper tip point using the 2D spline of the carapace as a reference geometry observed in Fig. 2e. This process ensured the flipper tip position was accurately positioned with respect to the turtle's carapace and time. After completion, a CAD part file was produced containing the data assembly. The process was repeated from seven separate sets of film, five sets from Fitzroy Island and the remainder from Heron Island, Australia. A similar process was applied to the front view film however, only four specimens were successfully obtained.

#### Data 3D conversion

**Figure 3:**
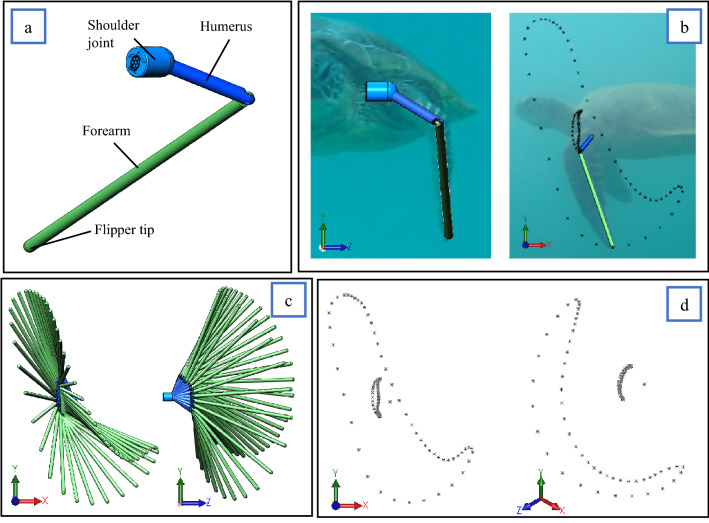
Data preparation. (**a**), Simplified sea turtle limb; (**b**), Rotation of limb in CAD, into 3D positions using 2-dimensional front and side data for reference; (**c**), Complete limb motion assembly viewed from xy and zy planes; (**d**), Conversion of limb motion into x,y,z data points of the flipper tip, elbow and shoulder rotation point, viewed from xy plane and an isometric viewpoint.

**Figure 4:**
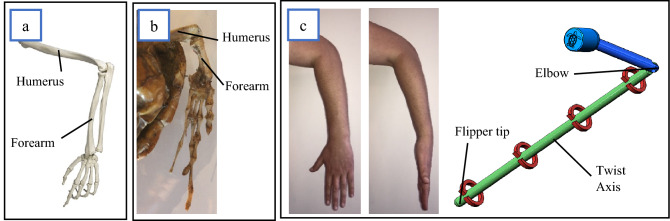
(**a**) Human arm skeleton (created by Henri et George/Shutterstock.com), (**b**) Hawksbill Sea turtle flipper skeleton. (**c**) Forearm rotation example.

Turning the 2D flipper paths into a 3D example required an iterative and repetitive process of assembling links representing the turtles' humerus (shoulder to elbow) and forearm (elbow to flipper tip) displayed in Fig. 3a. The links were modelled in Solidworks as individual part files ".SLDPRT". The size of each link was determined by identifying the position of the turtle's shoulder, elbow, and flipper tip from the side and frontal film with film images imported into CAD scaled to SCL of approximately 610 mm.To ensure the links modelled in CAD representing the humerus and forearm were anatomically correct in length, comparisons were made with a real sea turtle's skeletal anatomy, as seen in Fig. 4b. A Solidworks assembly was then created ".SLDASM." to assemble the links into a simplified 3D limb (Fig. 3a). The assembled limb could then be rotated in a SolidWorks assembly from the turtle's shoulder and elbow joints into position by simultaneously using the 2-dimensional data and the correspondent interval of the video footage from the side and front views to constrain the possible limb movement seen in Fig. 3b. This process was followed for each data point creating a full 3D representation of the turtle's stroke, as illustrated in Fig. 3c. From here, a new "SLDPRT" file could be generated consisting of the 3D data point with respect to time represented in Fig. 3d. These points consisted of the flipper tip, elbow and shoulder and were created by starting a "3D sketch" and mating each point concentrically to the joints and rotation point of interest from Fig. 3c.

#### Data postprocessing

The "SLDPRT" file containing all the data points along the x,y,z axis were exported as a ".igs" file type. This file type allows the data to be viewed and cleaned up in a simple text processing software (Notepad). The data was then imported into a numeric computing suite (Matlab) to have a "Fourier series" fitted with the x, y and z data represented by “Eqs. (1)–(3)”:

1 $$x\left(t\right) = {s}_{f}({a}_{x}+{\sum }_{i=1}^{n} {a}_{ix}\mathit{cos}\left(i{w}_{x}t\right)+ {b}_{ix}\mathit{sin}\left(i{w}_{x}t\right))$$

2 $$y\left(t\right) = {s}_{f}({a}_{y}+{\sum }_{i=1}^{n} {a}_{iy}\mathit{cos}\left(i{w}_{y}t\right)+ {b}_{iy}\mathit{sin}\left(i{w}_{y}t\right))$$

3 $$z\left(t\right) = {s}_{f}({a}_{z}+{\sum }_{i=1}^{n} {a}_{iz}\mathit{cos}\left(i{w}_{z}t\right)+ {b}_{iz}\mathit{sin}\left(i{w}_{z}t\right))$$

The Fourier coefficients were solved with the Matlab function "fourier8" to give the functions eight terms with the time variable defined as the frame rate used to collect the original data. A variable “${s}_{f}$” was added to the functions to allow a scaling factor to be applied to modify the functions to suit various sized turtles. Appling a “${s}_{f}$” equal to one was representative of a turtle with SCL equal to 610 mm and was defined as:

4 $${s}_{f}=\frac{{s}_{d}}{610}$$

where ${s}_{d}$ is the desired SCL in mm if scaling should be required. To understand the flipper rate of change in position and velocity, the position functions were differentiated with respect to time to create the flipper velocity, “Eq. (5)–(7)” and acceleration expressed as "Eq. (8)–(10)”:

5 $$\dot{x}\left(t\right) = {s}_{f}({\sum }_{i=1}^{n}-{i{w}_{x}a}_{ix}\mathit{sin}\left(i{w}_{x}t\right)+ i{w}_{x}{b}_{ix}\mathit{cos}\left(i{w}_{x}t\right))$$

6 $$\dot{y}\left(t\right) = {s}_{f}({\sum }_{i=1}^{n}-{i{w}_{y}a}_{iy}\mathit{sin}\left(i{w}_{y}t\right)+ i{w}_{y}{b}_{iy}\mathit{cos}\left(i{w}_{y}t\right))$$

7 $$\dot{z}\left(t\right) = {s}_{f}({\sum }_{i=1}^{n}-{i{w}_{z}a}_{iz}\mathit{sin}\left(i{w}_{z}t\right)+ i{w}_{z}{b}_{iz}\mathit{cos}\left(i{w}_{z}t\right))$$

8 $$\ddot{x}\left(t\right) = {s}_{f}({\sum }_{i=1}^{n}-{{\left(i{w}_{x}\right)}^{2}a}_{ix}\mathit{cos}\left(i{w}_{x}t\right)-{\left(i{w}_{x}\right)}^{2}{b}_{ix}sin\left(i{w}_{x}t\right))$$

9 $$\ddot{y}\left(t\right) = {s}_{f}({\sum }_{i=1}^{n}-{{\left(i{w}_{y}\right)}^{2}a}_{iy}\mathit{cos}\left(i{w}_{y}t\right)- {\left(i{w}_{y}\right)}^{2}{b}_{iy}sin\left(i{w}_{y}t\right))$$

10 $$\ddot{z}\left(t\right) = {s}_{f}({\sum }_{i=1}^{n}-{{\left(i{w}_{z}\right)}^{2}a}_{iz}\mathit{cos}\left(i{w}_{z}t\right)- {\left(i{w}_{z}\right)}^{2}{b}_{iz}\mathit{sin}\left(i{w}_{z}t\right))$$

#### Flipper twist model

The skeletal structure that supports the motion of the sea turtle flipper from a mechanical viewpoint is homologous to the human arm skeleton seen in Fig. 4a,b. For example, in Fig. 4c, it can be observed that the human forearm can create a twist from the elbow to the fingertips without any rotation of the humerus. Based on this assumption and from evidence observed in our video footage, we hypothesise that the flipper twist can be modelled to take place between the turtle elbow joint and flipper tip.

**Figure 5:**
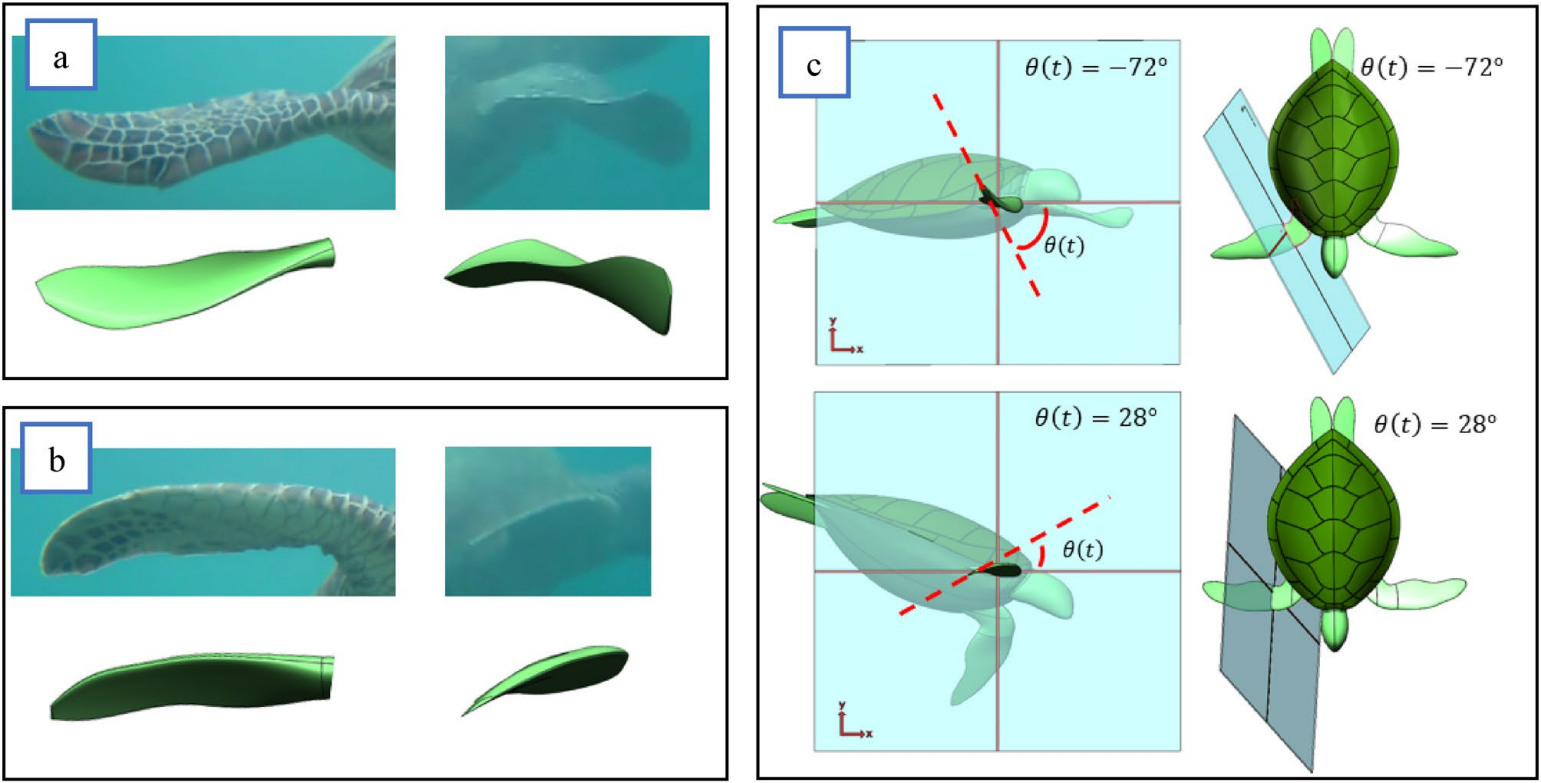
Flipper twist. (**a**), Downstroke twist of − 72°; (**b**), Upstroke twist of 28°; (**c**), Reference plane with 0° being inline with the horizontal x-axis of the elbow cross-section, additionally showing how the plane moves with the turtle flipper to remain normal to the rotation axis.

The twist angle $\theta (t)$ shown in Fig. 5c was defined using the right-hand rule based on the plane that intercepts the humerus and forearm with zero degrees in line with the horizontal x-axis of that plane. The amount of twist produced by the turtles was identified by comparing a detailed CAD geometry with the film images at various points along the upstroke and downstroke, as seen in Fig. 5a and b. The CAD geometry was twisted, keeping constant mass and volume until it closely matched the turtle's flipper in the film. The twisting of the CAD model was achieved using the Solidworks tool "Flex" by twisting the flipper geometry between a plane from the elbow to the flipper tip illustrated in Fig. 4c. The twist angle used was then numerically stored with respect to time for the postprocessing operations. Matlab was used in the same process as the previous section, fitting a function of flipper twist with respect to time. However, a piecewise function based on a Fourier series was used as "Eq. (11)”:

11 $$\theta \left(t\right)=\left\{\begin{array}{c}-72.4, 0\le t<0.84\\ {\sum }_{n=i}^{n}{a}_{dsi}\mathit{sin}({b}_{dsi}t+{c}_{dsi}), 0.84\le t<2.436\\ 27.9, 2.436\le t<3.612\\ {\sum }_{n=i}^{n}{a}_{usi}\mathit{sin}({b}_{usi}t+{c}_{usi}), 3.612\le t<4.2\end{array}\right.$$

12 $$\theta \left(x\right)=\frac{\theta \left(t\right)}{s}x$$

where $\theta (t)$ represents the angle of twist in degrees, the coefficients $a, b$ and $c$ are solved using the Matlab functions, “sin5” and “sin6” to give five and six terms to each function. The exact amount of flex on any point of the turtle's flipper could then be found applying “Eq. (12)”, where $x$ is the position of interest on the turtle wingspan $(s)$.

## Results

### Kinematics

#### Swimming pattern characteristics

Approximately one terabyte of film was collected during the period of observation. The suitable film sets were identified by sea turtles producing full locomotion pattern of the forelimb, as earlier described. Therefore, from the total amount of footage collected, we found only seven film sets were appropriate for side views, while four film sets were appropriated for front views. This selection process reduced the data from approximately one terabyte to two gigabytes and a total of eleven sets of film.

**Figure 6:**
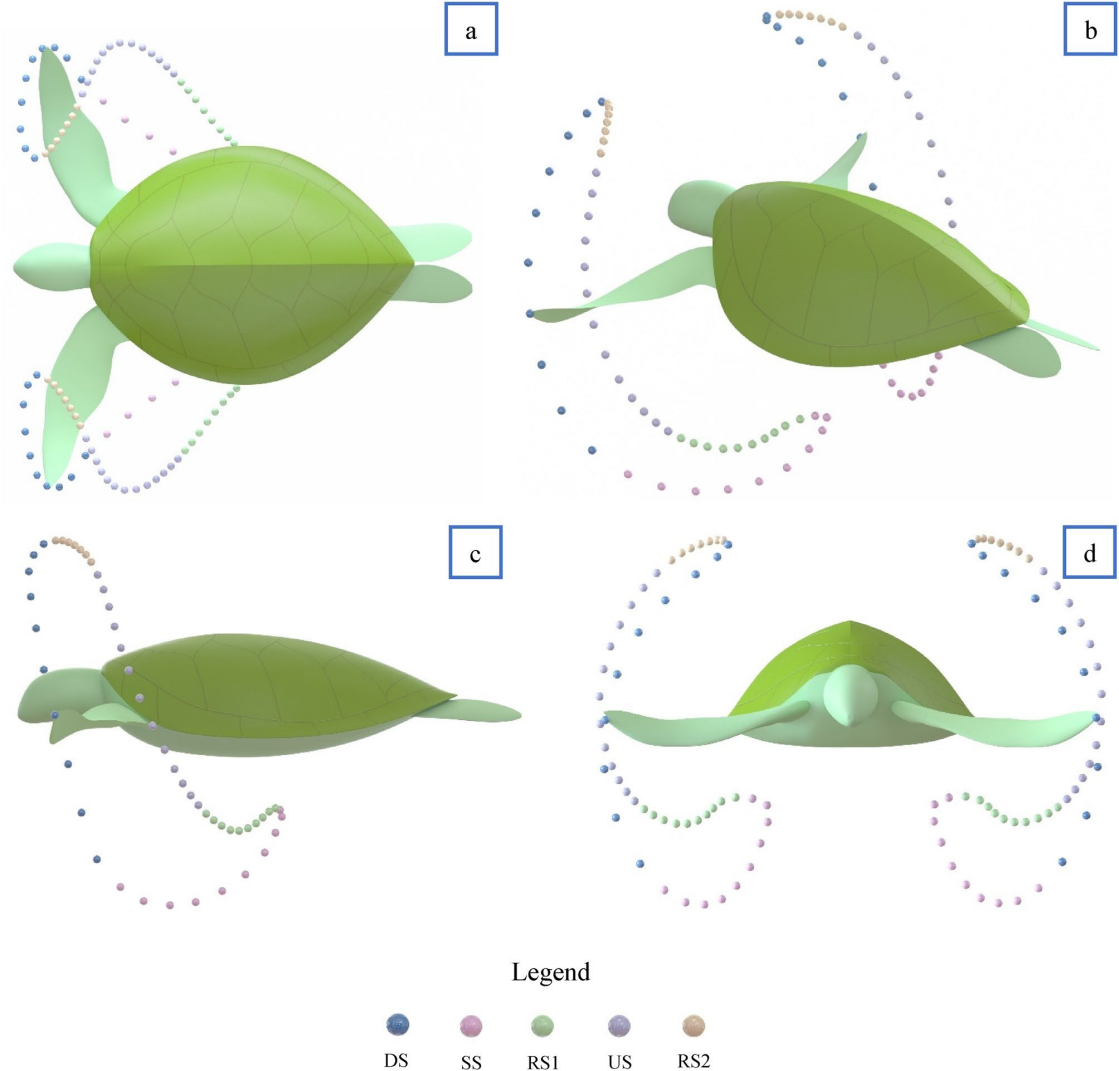
Complete 3D reconstruction of flipper tip motion from observational video. Data spheres represent 0.084 s time increments. When viewing from the Sagittal plane, the five stages can be characterised as follows: The downstroke (DS) is characterised by the fastest flipper motion with a slight reward motion. Sweep stroke (SS) is characterised by substantial horizontal displacement towards the centre of the carapace. Recovery stroke one (RS1) is characterised by slow-moving and modest horizontal displacement to recover the flipper into position for the upstroke. The upstroke (US) is characterised by a slow, predominantly straight path with slight forward motion. And finally, recovery stroke two (RS2) is characterised by the slowest moving section of the stroke to ready the flipper for the fast-moving downstroke. (**a**) View from Coronal plane. (**b**) 3D View, (**c**) View from Sagittal plane. (**d**) View from Transverse plane.

Observations showed that all turtles filmed (meaning the eleven above-mentioned selected sets) swam with the same general pattern as shown in Fig. 6, which resembles a closed loop with extended sweeping of the flipper tip towards the centre of the carapace from the Sagittal plane. At no time was the figure-of-eight pattern observed in the Sagittal plane previously reported by others^3–6^. However, the complete figure-of-eight pattern is evident from the Transverse and Coronal planes. The average limb beat cycle was 4.2 ± 0.32 s, closely matching observations from past research^25,30^. As observed in Fig. 6, we have broken down the flipper motion into five stages, “Downstroke (DS)”, “Sweep stroke (SS)”, “recovery stroke one (RS1)”, “upstroke (US)”, and “recovery stroke two (RS2)”. The stages are defined based on the flipper tip position in the Sagittal plane, as this was the only viewpoint that gave a clear line of sight to the flipper tip for all values of time. To help simplify comparing each of the turtle's swim patterns, the percentage of time spent in each of the five stages was compared. Table 1 outlines the time spent in each of the five stages from observed swimming patterns in wild animals. Our findings show that regardless of the turtle observed, limb beat frequency and ocean current, the flipper's percentage of time spent in each of the five motion stages was very similar.

**Table 1:** Amount of time the flipper tip spends in each of the five stages, data from the Sagittal plane.

Sea turtle film sets and location	DS (s)	SS (s)	RS1 (s)	US (s)	RS2 (s)	Limb beat cycle (s)
1 (Fitzroy Island)	0.9	0.8	0.9	1.1	0.6	4.3
2 (Fitzroy Island)	0.9	0.7	0.8	1.2	0.6	4.2
3 (Fitzroy Island)	1	1	1	1.3	0.7	5
4 (Fitzroy Island)	0.8	0.8	0.7	1.1	0.5	3.9
5 (Fitzroy Island)	0.7	0.7	0.8	1.1	0.4	3.7
6 (Heron Island)	0.7	0.9	0.7	1.2	0.9	4.4
7 (Heron Island)	0.8	0.7	0.7	1.2	0.5	3.9

Refer to Supplementary Fig. S1 for swimming patterns.

#### General swimming model

**Figure 7:**
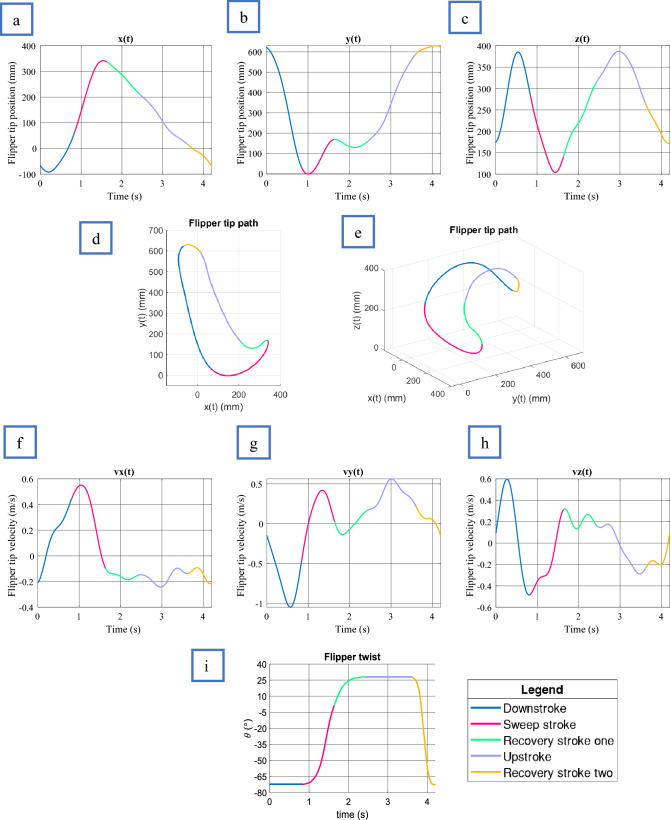
Forelimb flipper kinematics plotted against time. Shoulder rotation point located at (*92.5, 305, 27.6*) (mm). All plots represent a turtle of SCL equal to 610 mm. (**a**) Flipper tip position in x, (**b**) Flipper tip position in y, (**c**) Flipper tip position in z, (**d**) Flipper tip path in x, y plane. (**e**) Flipper tip path 3D. (**f**) Flipper tip velocity in x, (**g**) Flipper tip velocity in y, (**h**) Flipper tip velocity in z, (**i**) Flipper twist.

**Figure 8:**
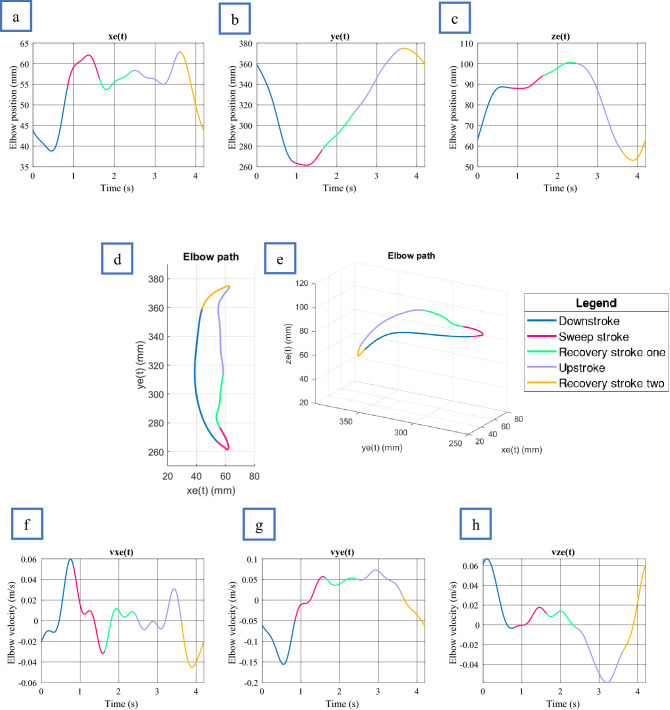
Elbow kinematics plotted against time. Shoulder rotation point located at (92.5, 305, 27.6) (mm). All plots represent a turtle of SCL equal to 610 mm. (**a**) Flipper elbow position in x, (**b**) Flipper elbow position in y, (**c**) Flipper elbow position in z, (**d**) Flipper elbow path in x, y plane. (**e**) Flipper elbow path 3D. (**f**) Flipper elbow velocity in x, (**g**) Flipper elbow velocity in y, (**h**), Flipper elbow velocity in z.

This section will continuously refer to Fig. 7 and 8, which details the flippers five stages of motion. All plots are representative of a turtle with SCL equal to 610 mm and “${s}_{f}$” equal to one. During the “DS”, the flipper tip and elbow roll down together, creating the fastest part of the limb beat. The flipper twist during the DS is at its most aggressive average angle of − 72.4 ± 2.5º, with the elbow going through its most significant displacement during this time. As viewed from the Sagittal plane, the flipper tip takes a mostly straight path, making up 19.7% ± 1.4% of the limb cycle. The “SS” movement starts when the humerus begins to slow, and the elbow accelerates the forelimb in a curved sweeping motion until the flipper tip reaches approximately half the carapace length. During this stroke section, the flipper twist rotates to 0°, bringing the two flippers parallel beneath the carapace. It can be observed that the elbow begins to rise before the forelimb reaches the end of the SS, creating a clapping motion similar to sea lions^31^. Overall the SS made up 19% ± 1.1% of the limb cycle. “RS1” is a slow-moving motion of the humerus and forelimb to allow the turtle to get itself ready for the upstroke by flexing the forelimb to an average value of 27.9 ± 4.8°. Overall the RS1 made up 19% ± 1.5% of the overall limb cycle. The “US” occurred at approximately 50% of the downstroke speed, matching previous findings^3^. During the entire US, a steady flipper average twist of 27.9 ± 4.8° was observed. Overall, the US made up 28% ± 1.4% of the limb cycle and looked entirely passive in motion. The final section, “RS2”, is the slowest moving of the limb beat cycle. During this process, the flipper twist travels through its most extensive Rotation from 27.9 ± 4.8° to − 72.4 ± 2.5°, making up 14% ± 2.2% of the overall limb cycle. The possible reason for the slow movement of RS2 is to help reduce turbulence during the aggressive change in flipper twist, thus potentially lowering the drag coefficient.

#### Result verification

**Figure 9:**
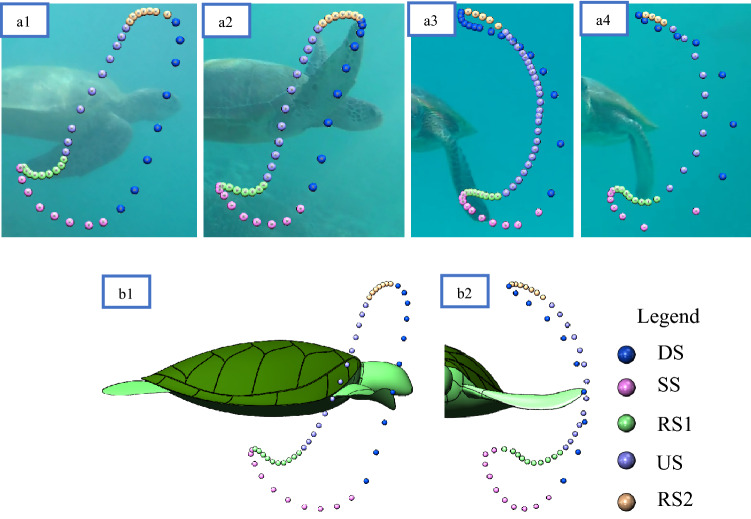
Comparison of various sea turtles filmed at different locations and times compared with the proposed three-dimensional model. (**a**) Observed Flipper paths from four different sea turtle specimens spheres within the figure representing the position of the flipper tip at 0.1-s increments. (**b**) The proposed 3D model flipper path with spheres within the figure represents the flipper tip's position at 0.084-s increments.

As the combination of separate sets of two-dimensional data creates uncertainty in the model, we help verify the results by graphically comparing our three-dimensional model with various footage sets of swimming turtles. Such verification shows a good visual correlation between all footage sets (Fig. 9a) and our model (Fig. 9b). As displayed in Fig. 9a, in four different specimens (Fig. 9a.1–a.4), though the flipper paths slightly differed from one turtle to the other, excellent correlations were observed between the five flipper stages, with the general swimming pattern evident in all specimens that produced complete wing oscillations. For instance, the blue spheres representing the downstroke, it can be observed this produces the fastest section of the limb cycle, with each sphere representing 0.1-s time intervals for Fig. 9a. Additionally, the downstroke can be seen producing a small reward horizontal motion. The pink spheres producing the sweep stroke can be observed in all specimens to produce large amounts of horizontal displacement in Fig. 9a.1 and a.2, with a clapping motion as seen in Fig. 9a.3 and a.4. Recovery stroke one shown in green spheres can be observed in all specimens with considerable slow-moving horizontal displacement to recover the flipper into a position suitable for the upstroke. The upstroke shown in purple spheres can be seen in all specimens to produce a slow vertical motion with low levels of forwards motion. As seen in Fig. 9a.3 and a.4, the upstroke crosses paths with the downstroke to create a figure-of-eight path before finally arriving at recovery stroke two, where the flipper travels at low speed into a position suitable for the aggressive downstroke. Additionally, a “MATLAB” program (available in supplementary documents under: turtle_flipper.m with precompiled animations shown in Figs. S2–S4) was created to verify the flipper's motion. The program solves the three-dimensional model to produce a live three-dimensional animation of the flipper motion giving illustrative graphical feedback, where the model presented in this work is visually compared with the flipper motion of wild green sea turtles.

## Discussion

We present a five-stage cycle swimming locomotion model, in contrast to the previous four-stage ones^5,6^, adding a new finding we have named the sweep stroke. Based on findings from previous work, this model could also potentially represent the swimming of the Hawksbill^4^ and Loggerhead turtle^20^. Our results provided no evidence of a figure-of-eight pattern in the sagittal plane previously reported^3–6^. Instead, it showed a closed-loop with extended sweeping of the flipper tip towards the centre of the carapace to create a swept clapping motion. However, a figure-of-eight pattern is evident in the transverse and coronal planes. Although our methods for creating the three-dimensional models have been generated from two-dimensional data, thus creating what could be called the best guess, we have illustrated an excellent correlation between the model compared with natural swimming footage. Additionally, the advantages of this process of collecting the data from the animal's natural habitat without disturbing the animal far outweigh any disadvantages compared to more traditional data collection methods. This could potentially lead to other animals being analysed by filming with non-invasive underwater drone technologies. The model presented here could lead to a better comprehension of the sea turtle propulsion methods and their fluid–structure interaction.

## Supplementary Information

Supplementary Figures.

Supplementary Information 2.

Supplementary Information 3.

## Data Availability

All data sets for the current study are available from the authors on reasonable request, via contacting correspondence author.

## References

[1] Chiari Y, Cahais V, Galtier N, Delsuc F (2012). Phylogenomic analyses support the position of turtles as the sister group of birds and crocodiles (Archosauria). BMC Biol..

[2] Hochscheid S, Bentivegna F, Speakman JR (2003). The dual function of the lung in chelonian sea turtles: Buoyancy control and oxygen storage. J. Exp. Mar. Biol. Ecol..

[3] Davenport J, Munks SA, Oxford PJ (1984). A comparison of the swimming of marine and freshwater turtles. R. Soc. Lond. B.

[4] Walker WF (1971). Swimming in sea turtles of the family Cheloniidae. Copeia.

[5] Font D (2011). Design and implementation of a biomimetic turtle hydrofoil for an autonomous underwater vehicle. Sensors.

[6] Xu, J., Liu, X., Chu, D., Sun, L. & Zhang, M. in *2009 IEEE International Conference on Robotics and Biomimetics (ROBIO).*

[7] Eckert SA (2006). High-use oceanic areas for Atlantic leatherback sea turtles (*Dermochelys coriacea*) as identified using satellite telemetered location and dive information. Mar. Biol..

[8] Hays GC, Scott R (2013). Global patterns for upper ceilings on migration distance in sea turtles and comparisons with fish, birds and mammals. Funct. Ecol..

[9] Luschi P, Hays GC, Del Seppia C, Marsh R, Papi F (1998). The navigational feats of green sea turtles migrating from Ascension Island investigated by satellite telemetry. Proc. R. Soc. Lond. B.

[10] Izraelevitz JS, Triantafyllou MS (2014). Adding in-line motion and model-based optimisation offers exceptional force control authority in flapping foils. J. Fluid Mech..

[11] Licht SC, Wibawa MS, Hover FS, Triantafyllou MS (2010). In-line motion causes high thrust and efficiency in flapping foils that use power downstroke. J. Exp. Biol..

[12] Zhou K, Liu J-K, Chen W-S (2018). Numerical and experimental studies of hydrodynamics of flapping foils. J. Hydrodyn..

[13] Chen L, Wu J, Cheng B (2020). Leading-edge vortex formation and transient lift generation on a revolving wing at low Reynolds number. Aerospace Sci. Technol..

[14] Jardin T (2017). Coriolis effect and the attachment of the leading edge vortex. J. Fluid Mech..

[15] Lu XY, Yang JM, Yin XZ (2003). Propulsive performance and vortex shedding of a foil in flapping flight. Acta Mech..

[16] Depecker M, Broin FDLD, Renous S, Davenport J, Bels V (2007). Biology of Turtles.

[17] Renous S, Bels V, Davenport J (2000). Locomotion in marine Chelonia: Adaptation to the aquatic habitat. Hist. Biol..

[18] Rivera ARV, Wyneken J, Blob RW (2011). Forelimb kinematics and motor patterns of swimming loggerhead sea turtles (*Caretta caretta*): Are motor patterns conserved in the evolution of new locomotor strategies?. J. Exp. Biol..

[19] Renous S, Bels V (1993). Comparison between aquatic and terrestrial locomotions of the leatherback sea turle (*Dermochelys coriacea*). J. Zool..

[20] Rivera AR, Rivera G, Blob RW (2013). Forelimb kinematics during swimming in the pig-nosed turtle, *Carettochelys insculpta*, compared with other turtle taxa: Rowing versus flapping, convergence versus intermediacy. J. Exp. Biol..

[21] Cieri RL, Hatch ST, Capano JG, Brainerd EL (2020). Locomotor rib kinematics in two species of lizards and a new hypothesis for the evolution of aspiration breathing in amniotes. Sci. Rep..

[22] Weller HI (2020). An XROMM study of food transport and swallowing in channel catfish. Integr. Org. Biol..

[23] Smolowitz RJ, Patel SH, Haas HL, Miller SA (2015). Using a remotely operated vehicle (ROV) to observe loggerhead sea turtle (*Caretta caretta*) behavior on foraging grounds off the mid-Atlantic United States. J. Exp. Mar. Biol. Ecol..

[24] Maki T, Horimoto H, Ishihara T, Kofuji K (2020). Tracking a sea turtle by an AUV with a multibeam imaging sonar: Toward robotic observation of marine life. Int. J. Control Autom. Syst..

[25] Kinoshita C, Fukuoka T, Narazaki T, Niizuma Y, Sato K (2021). Analysis of why sea turtles swim slowly: A metabolic and mechanical approach. J. Exp. Biol..

[26] Prange HD (1976). Energetics of swimming of a sea turtle. J. Exp. Biol..

[27] Watanabe YY (2011). Scaling of swim speed in breath-hold divers. J. Anim. Ecol..

[28] Narazaki T, Sato K, Abernathy KJ, Marshall GJ, Miyazaki N (2009). Sea turtles compensate deflection of heading at the sea surface during directional travel. J. Exp. Biol..

[29] Narazaki T, Sato K, Abernathy KJ, Marshall GJ, Miyazaki N (2013). Loggerhead turtles (*Caretta caretta*) use vision to forage on gelatinous prey in mid-water. PLoS ONE.

[30] Hays GC, Metcalfe JD, Walne AW, Wilson RP (2004). First records of flipper beat frequency during sea turtle diving. J. Exp. Mar. Biol. Ecol..

[31] Kashi E, Kulkarni AA, Perrotta G, Leftwich MC (2020). Flowfields produced by a robotic sea lion foreflipper starting from rest. Bioinspir. Biomim..

