# Supplementary material for: New insight into the swimming kinematics of wild Green sea turtles (Chelonia mydas)
Source: Sci Rep. 2022 Oct 31;12:18151. doi: 10.1038/s41598-022-21459-y (PMC9622894; doi:10.1038/s41598-022-21459-y)
Supplement: Supplementary file 1 — Supplementary Figures. [file 41598_2022_21459_MOESM1_ESM.pptx]

## Slide 1
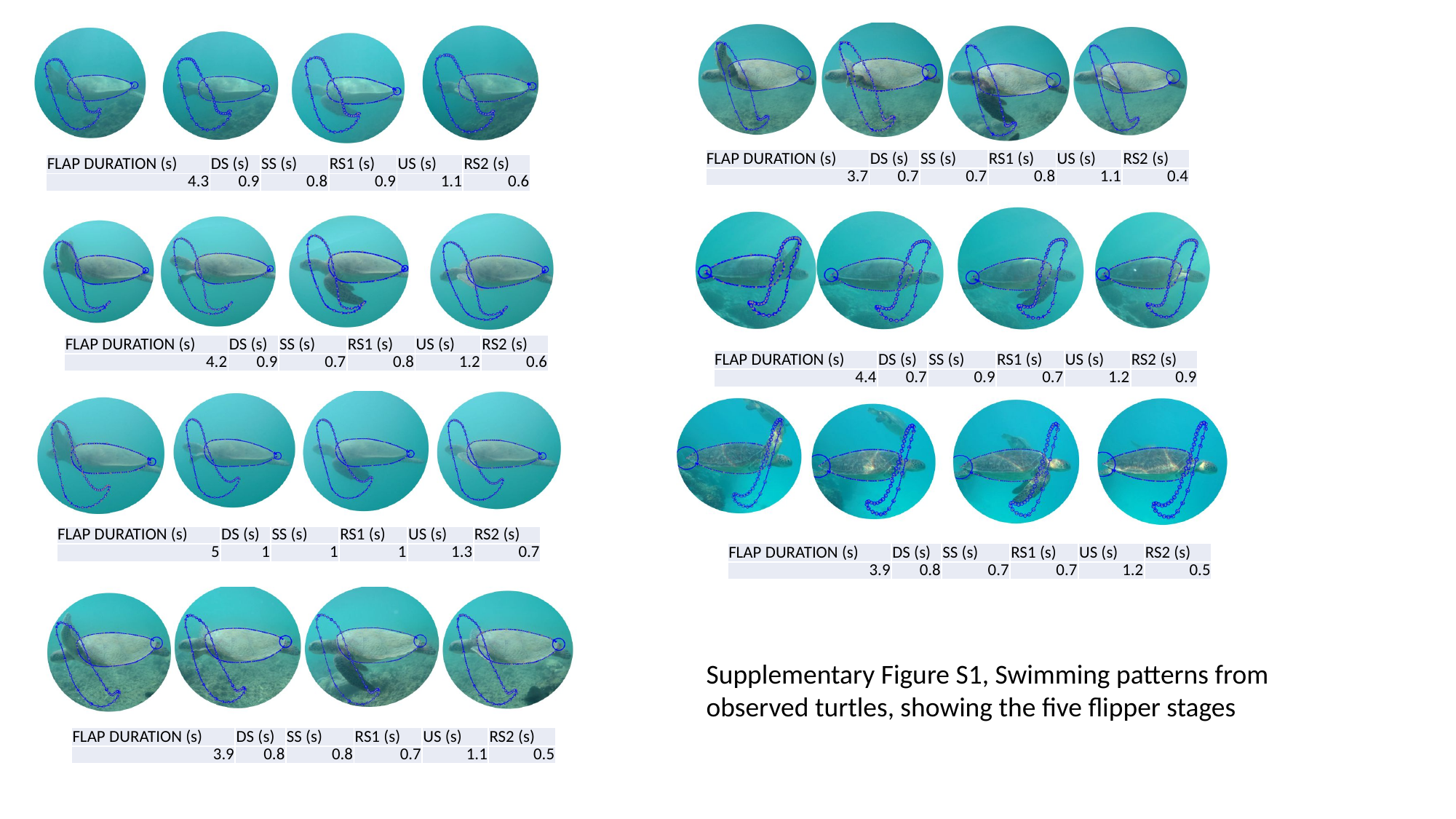

| FLAP DURATION (s) | DS (s) | SS (s) | RS1 (s) | US (s) | RS2 (s) |
| --- | --- | --- | --- | --- | --- |
| 3.7 | 0.7 | 0.7 | 0.8 | 1.1 | 0.4 |
| FLAP DURATION (s) | DS (s) | SS (s) | RS1 (s) | US (s) | RS2 (s) |
| --- | --- | --- | --- | --- | --- |
| 4.3 | 0.9 | 0.8 | 0.9 | 1.1 | 0.6 |
| FLAP DURATION (s) | DS (s) | SS (s) | RS1 (s) | US (s) | RS2 (s) |
| --- | --- | --- | --- | --- | --- |
| 4.2 | 0.9 | 0.7 | 0.8 | 1.2 | 0.6 |
| FLAP DURATION (s) | DS (s) | SS (s) | RS1 (s) | US (s) | RS2 (s) |
| --- | --- | --- | --- | --- | --- |
| 4.4 | 0.7 | 0.9 | 0.7 | 1.2 | 0.9 |
| FLAP DURATION (s) | DS (s) | SS (s) | RS1 (s) | US (s) | RS2 (s) |
| --- | --- | --- | --- | --- | --- |
| 5 | 1 | 1 | 1 | 1.3 | 0.7 |
| FLAP DURATION (s) | DS (s) | SS (s) | RS1 (s) | US (s) | RS2 (s) |
| --- | --- | --- | --- | --- | --- |
| 3.9 | 0.8 | 0.7 | 0.7 | 1.2 | 0.5 |
Supplementary Figure S1, Swimming patterns from observed turtles, showing the five flipper stages
| FLAP DURATION (s) | DS (s) | SS (s) | RS1 (s) | US (s) | RS2 (s) |
| --- | --- | --- | --- | --- | --- |
| 3.9 | 0.8 | 0.8 | 0.7 | 1.1 | 0.5 |

## Slide 2
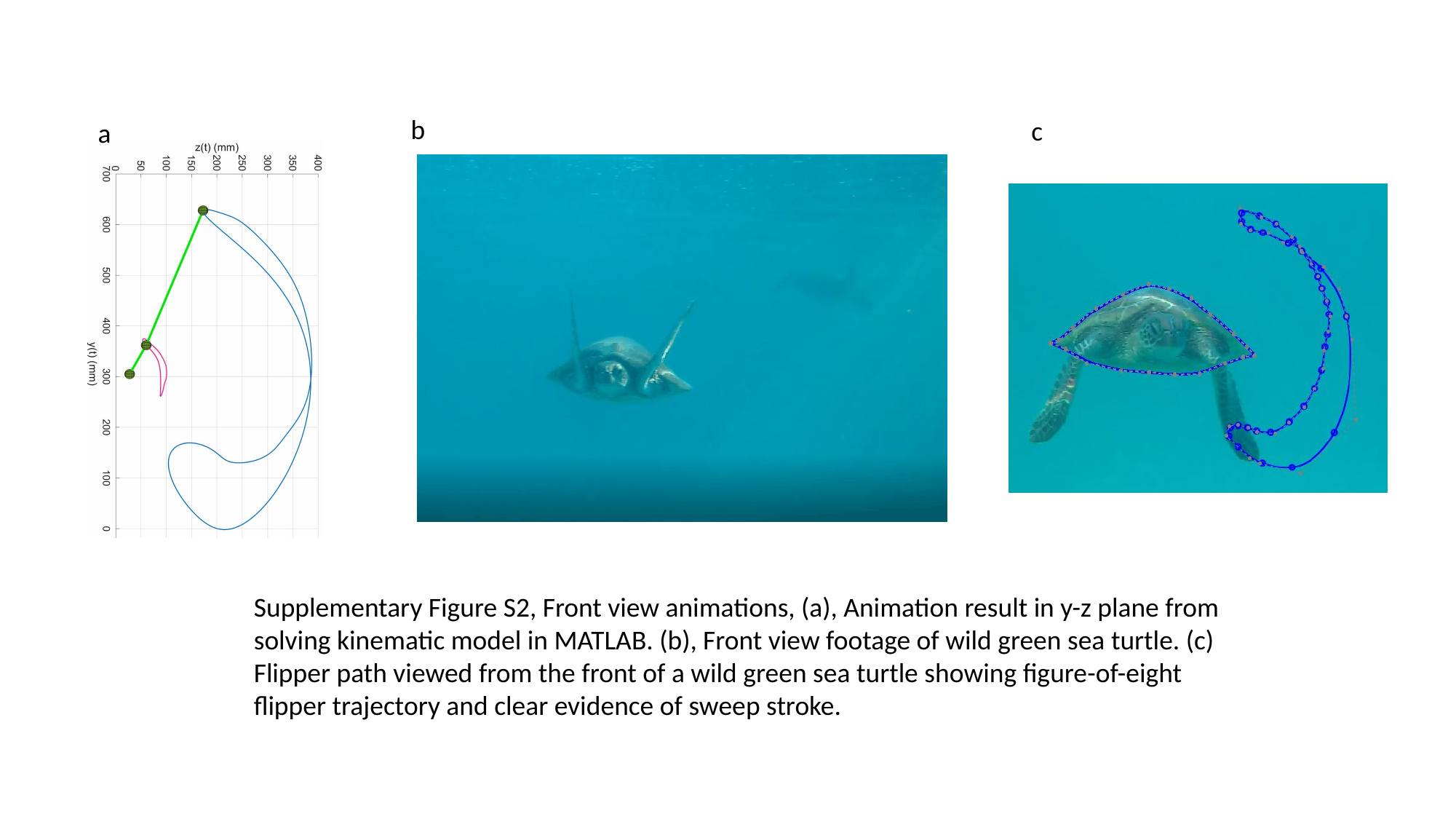

b
c
a
Supplementary Figure S2, Front view animations, (a), Animation result in y-z plane from solving kinematic model in MATLAB. (b), Front view footage of wild green sea turtle. (c) Flipper path viewed from the front of a wild green sea turtle showing figure-of-eight flipper trajectory and clear evidence of sweep stroke.

## Slide 3
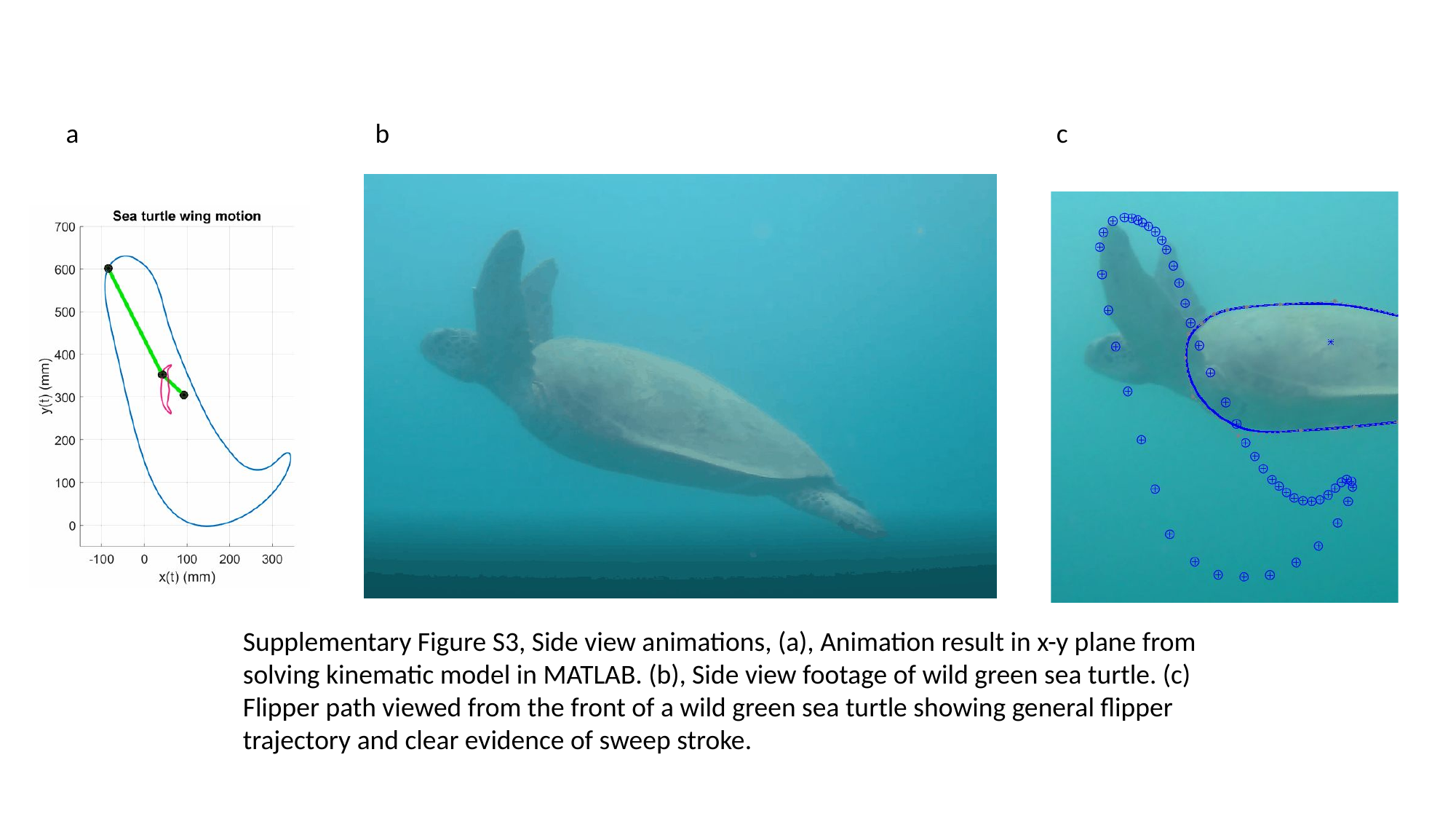

a
b
c
Supplementary Figure S3, Side view animations, (a), Animation result in x-y plane from solving kinematic model in MATLAB. (b), Side view footage of wild green sea turtle. (c) Flipper path viewed from the front of a wild green sea turtle showing general flipper trajectory and clear evidence of sweep stroke.

## Slide 4
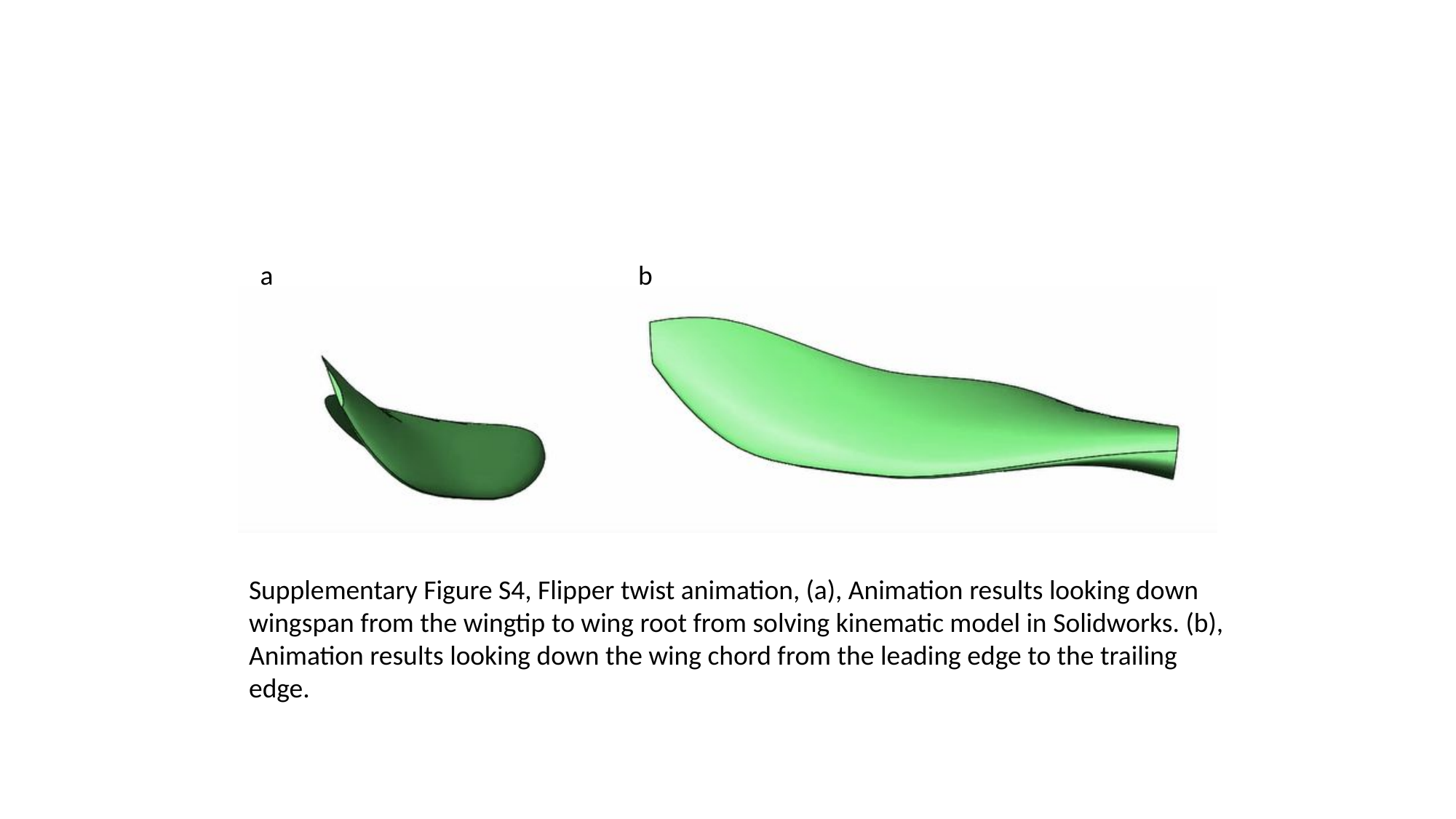

a
b
Supplementary Figure S4, Flipper twist animation, (a), Animation results looking down wingspan from the wingtip to wing root from solving kinematic model in Solidworks. (b), Animation results looking down the wing chord from the leading edge to the trailing edge.
