# Supplementary material for: New insight into the swimming kinematics of wild Green sea turtles (Chelonia mydas)
Source: Sci Rep. 2022 Oct 31;12:18151. doi: 10.1038/s41598-022-21459-y (PMC9622894; doi:10.1038/s41598-022-21459-y)
Supplement: Supplementary file 2 — Supplementary Information 2. [file 41598_2022_21459_MOESM2_ESM.pdf]

Title: Supplementary Documents of: *New insight into the swimming kinematics of wild Green sea turtles (Chelonia mydas).*

Supplementary Documents contain:

1- **Animations**

This pptx file contains animations comparing wild sea turtle swimming patterns against kinematic model.

- Supplementary Figure S1: Swimming patterns from observed turtles, showing the five flipper stages.
- Supplementary Figure S2: Front view animations, (a), Animation result in y-z plane from solving kinematic model in MATLAB. (b), Front view footage of wild green sea turtle. (c) Flipper path viewed from the front of a wild green sea turtle showing figure-of-eight flipper trajectory and clear evidence of sweep stroke.
- Supplementary Figure S3: Side view animations, (a), Animation result in x-y plane from solving kinematic model in MATLAB. (b), Side view footage of wild green sea turtle. (c) Flipper path viewed from the front of a wild green sea turtle showing general flipper trajectory and clear evidence of sweep stroke.
- Supplementary Figure S4: Flipper twist animation, (a), Animation results looking down wingspan from the wingtip to wing root from solving kinematic model in Solidworks. (b), Animation results looking down the wing chord from the leading edge to the trailing edge.

2- **Turtle flipper.m**

This file provides the Matlab Code to solve the kinematics and display the animations as shown in Fig S1- S2

Any additional data set or CAD files are available on reasonable request via direct contact to:

Dr Lorenzo Garcia

lorenzo.garcia@aut.ac.nz

BioDesign Lab

Auckland University of Technology

New Zealand
